# Lung cancer incidence and mortality in trend and prediction between 2012-2030 in Shandong Province, using a Bayesian age-period-cohort model

**DOI:** 10.3389/fonc.2024.1451589

**Published:** 2024-12-04

**Authors:** Fan Jiang, Zhentao Fu, Jie Chu, Jie Ren, Chunxiao Xu, Xiaohui Xu, Xiaolei Guo, Zilong Lu, Aiqiang Xu

**Affiliations:** ^1^ Department of Chronic and Non-communicable Disease Control and Prevention, Shandong Center for Disease Control and Prevention, Jinan, China; ^2^ Institute of Preventive Medicine in Shandong University, Shandong Academy of Preventive Medicine, Jinan, China

**Keywords:** lung cancer, incidence, mortality, prediction, Shandong

## Abstract

**Objectives:**

Lung cancer is one of the most common cancers in Shandong Province, China. Projecting future cancer trend is crucial for planning cancer control. We aimed to examine the trend of lung cancer incidence and mortality from 2012 to 2023, and predict the lung cancer burden to 2030 in Shandong.

**Methods:**

Data of lung cancer incidence and mortality from 2012 to 2023 were obtained from the Shandong Cancer Registries. The average annual percentage change (AAPC) was used to quantify the trend of the lung cancer age-standardised rate using Joinpoint software. Bayesian age-period-cohort model was used to predict lung cancer incidence and mortality from 2024 to 2030.

**Results:**

The age-standardised incidence rate (ASIR) remained stable from 2012 to 2023. The ASIR in males decreased with an AAPC of -1.350%, while the ASIR in females increased with an AAPC of 2.429%. The age-standardised mortality rate (ASMR) decreased with an AAPC of -2.911%. This trend was also observed in males (AAPC=-2.513%), females (AAPC=-3.632%), urban areas (AAPC=-3.267%) and rural areas (AAPC=-2.603%). For our predictions, the ASIR will increase to 49.21 per 100,000 until 2030, with an AAPC of 1.873%. This upward trend is expected for females and urban areas, with an AAPC of 4.496% and 4.176%, while it is not observed for males and rural areas. The ASMR is expected to remain stable up to 2030, and this trend will maintain both in males and females. The ASMR will exhibit an upward trend (AAPC=1.100%) in urban areas and a downward trend (AAPC=-0.915%) in rural areas.

**Conclusion:**

The ASIR of lung cancer will increase until 2030, while the ASMR of lung cancer is expected to remain stable in Shandong. It is necessary to take further preventive measures such as strengthening tobacco control, enhancing health education and expanding screening efforts.

## Introduction

Lung cancer has been the most commonly cancer and the leading cause of cancer death worldwide. According to the latest global cancer statistics estimates in 2022, there have been about 2.48 million new cases of lung cancer in the world, accounting for 12.4% of the total new cases, and about 1.82 million lung cancer deaths, accounting for 18.7% of the total cancer deaths (1). In China, lung cancer has always ranked first in terms of incidence and mortality, with 1.06 million new cases of lung cancer and 0.73 million lung cancer deaths based on the Chinese cancer registry data statistics in 2022 (2).

Since the 1960s, developed countries began to implement tobacco control measures in time to control the growth of lung cancer (3). In the United States, the incidence of lung cancer in men has been decreasing since its peak in 1980s, while the incidence of lung cancer in women has been decreasing since 2005 (4). Nevertheless, in China, the incidence and mortality of lung cancer have increased rapidly in the past 30 years, with an annual increase of 3.7% and 3.3%, respectively. Until in the last decade, the age-standardised incidence rate (ASIR) reached a plateau while the age-standardised mortality rate (ASMR) showed a slight downward trend (5). As the increasing ageing world population, the disease burden of lung cancer will be likely to continue to increase in developing countries especially in China.

Shandong is the second most populous province in China, with 102 million people, accounting for 7.2% of the Chinese population. The large population base leads to large numbers of lung cancer cases and deaths, and the disease burden of lung cancer in Shandong is very heavy. According to data from the Shandong Cancer Registries (6), in 2018, the ASIR and ASMR of lung cancer in Shandong were 42.56 per 100,000 and 29.77 per 100,000 respectively, which were higher than the ASIR and ASMR in China (38.23 per 100,000 and 27.18 per 100,000) (7). In addition, Shandong is one of the provinces with the fastest growth rate in lung cancer incidence and mortality in China (8, 9), and the disease burden of lung cancer was also significantly higher than other provinces in China, such as Henan Province (10), Jiangsu Province (11), Sichuan Province (12), Gansu Province (13) and Jiangxi Province (14). Therefore, understanding and predicting the epidemic trend of lung cancer in Shandong can provide an important basis for the study of lung cancer prevention and control strategies, so as to effectively reduce the burden of lung cancer in Shandong.

Through mathematical models, future cancer burden can be predicted using past surveillance data, based on the assumption that recent incidence or mortality trends will continue to some extent in the future. In particular, Bayesian age-period-cohort model (BAPC) has demonstrated its efficacy as a tool for analysing and predicting incidence and mortality trends (15, 16). In the UK, this model was used to predict cancer incidence and mortality in the country in 2020, 2025, 2030 and 2035 (17–20). Scholars in our country have also used this model to predict the mortality of esophageal cancer and the incidence of lung cancer (21, 22). Currently, few studies were reported on predicting the burden of lung cancer based on population cancer registration data in Shandong Province and even in China. In view of this, our study aimed to provide an estimate of the burden of lung cancer through 2030, to provide the reference basis for formulating the accurate prevention and control policy of lung cancer.

## Materials and methods

### Data sources

The Shandong Center for Disease Control and Prevention takes on the crucial role of assembling, assessing, and examining cancer-related information from population-based registries. Data for cancer patients was comprehensively furnished by cancer registries in Shandong, which encompassed 13 urban and 22 rural registries in 2023. Lung cancer cases were defined, according to the tenth revision of the International Statistical Classification of Diseases (23), by code C33-C34. The final dataset included variables describing year, sex, age at diagnosis, death age, region (urban or rural area), diagnosis date, death date. Age was divided into 18 subgroups, including 0-4 years, 5-9 years, 10-14 years, and then in 5-year age groups up to 80-84 years, and finally 85 years or older. The population data by sex and age group came from the Census Register of Public Security and the Statistical Yearbook in Shandong. All lung cancer cases in Shandong between 2012 and 2023 were included.

### Quality control

The quality of the cancer registration data was assessed according to the standards and requirements of “Guideline for Chinese Cancer Registration (2016)” and International Agency for Research on Cancer/International Association of Cancer Registries (IARC/IACR), including the validity, reliability, completeness and comparability (24, 25). The key indicators for quality control include the mortality to incidence ratio (M/I), the proportion of morphological verification (MV%), and the percentage of cases identified with death certification only (DCO%). Eligible data from cancer registries in 2023 covered 37.46 million people, accounting for 37.52% of the Shandong Province population.

### Statistical analysis

Lung cancer data was summarised and analysed using SAS (version 9.4) and Excel (version 2013). We obtained statistics including incidence, age-specific incidence, mortality and age-specific mortality of lung cancer calculated by year, sex, urban and rural areas. The age-standardised rate was adjusted based on the age composition of Chinese standard population in 2000. To assess the overall trends across multiple periods comprehensively, the average annual percentage change (AAPC) was employed to measure the temporal progression of incidence and mortality rates (26). This method captured the rates from 2012 to 2023, reflecting past trends, and from 2024 to 2030, signifying future trends. The AAPC and 95% confidence intervals (CI) were estimated by Joinpoint (version 4.8.0.1). A positive AAPC indicates an upward trend in incidence (mortality) over this time period, whereas a downward trend if the AAPC is negative (27).

Age-period-cohort model can analyse the effect of age, period and cohort on cancer incidence and mortality, and predict the incidence and mortality according to the effect value of each factor. The classical age-period-cohort model is general linear model. When only age and period factors are included in the model, it is called an age-period model. When only age and cohort factors are included in the model, it is called an age-cohort model. The Epi package in R software can build age-period-cohort model and choose the best model by comparing the deviance of different models. After identifying the factors that need to be incorporated into the model, the model can be used for predictive analysis.

This model was implemented using the Bayesian Age-Period-Cohort Modelling and Prediction package (BAMP) of R (version 4.2.3) (28). Bayesian method can use not only the information of the sample, but also other known information outside the sample, that is, the prior information. The Bayesian method provides a way to calculate the probability of a hypothesis by combining prior information about an unknown parameter with sample information according to a Bayesian formula, then the unknown parameters are inferred according to the posterior information (29). BAMP describes the effect of age, period, and cohort by random walk (RW) priors of different orders (30). The RW-1 prior assumes a constant trend over the time scale, whereas the RW-2 prior assumes a linear time trend (31, 32). The results of the iterations were used to estimate the parameter values for age, period, and cohort effects based on different RW choices through Markov chain Monte Carlo (MCMC) method iterations. The more iterations, the higher the accuracy of the model. This method can smooth the effect of age, period and cohort, and avoid the large fluctuation between the two groups, so that the estimation result is more stable and reliable (33).

In this study, we first observed the change trend of period and cohort factors of lung cancer incidence and mortality using the function of apc.fit and apc.frame in Epi package, and selected the suitable RW combination incorporating the BAMP model. MCMC simulations were run for 1,010,000 iterations with the initial 10,000 iterations used as burn-in to minimize the effect of initial values. The median iterative values and 95% CI (using 2.5% and 97.5% of the 1,000,000 iterated results, respectively) were obtained by the MCMC simulations in the models. Finally, we obtained the predictions of lung cancer incidence and mortality in 2024-2030. The posterior deviance and predictive deviance of the model were used as a measure of the goodness of fit.

## Result

### Lung cancer incidence and mortality in Shandong, 2012-2023

**Table 1** showed the lung cancer incidence in Shandong from 2012 to 2023. The crude incidence rate of lung cancer showed a significant upward trend, increasing from 66.96 per 100,000 to 85.34 per 100,000 (AAPC=2.295%, *P*<0.01). The ASIR remained stable for the 12-year period. For both males and females, the crude incidence rate displayed an increasing trend, with an AAPC of 1.254% (*P*=0.01) in males and an AAPC of 4.078% (*P*<0.01) in females. The ASIR in males decreased from 58.59 per 100,000 to 51.34 per 100,000 (AAPC=-1.350%, *P*<0.01), while the ASIR in females increased from 28.61 per 100,000 to 35.71 100,000 (AAPC=2.429%, *P<*0.01). The crude incidence rate showed an increasing trend in rural areas (AAPC=3.549%, *P*<0.01) but no significant change in urban areas. The ASIR remained stable both in urban and rural areas.

**Table 1:** Incidence of lung cancer from 2012 to 2023 in Shandong (per 100,000).

Year	Overall		Male		Female		Urban		Rural	
	Crude Rate	ASIR	Crude Rate	ASIR	Crude Rate	ASIR	Crude Rate	ASIR	Crude Rate	ASIR
2012	66.96	43.00	86.59	58.59	46.96	28.61	65.96	41.45	67.90	44.50
2013	68.05	43.21	87.80	58.80	47.92	28.45	67.84	41.56	68.23	44.62
2014	72.60	43.86	92.95	59.23	51.82	29.52	71.21	41.34	73.75	46.01
2015	70.57	41.28	92.50	57.15	48.16	26.58	74.61	40.52	68.48	41.83
2016	75.04	41.55	97.31	57.03	52.30	27.29	78.39	40.86	72.57	42.11
2017	75.59	41.79	96.54	55.91	54.24	28.68	74.36	39.58	76.42	43.37
2018	78.15	42.56	97.55	55.24	58.43	30.83	77.74	40.83	78.49	44.22
2019	85.35	45.47	103.84	57.03	66.59	34.70	82.46	43.08	86.54	46.91
2020	89.45	46.87	108.36	58.47	70.11	36.14	88.02	45.32	90.50	48.08
2021	79.97	41.77	94.38	50.77	65.35	33.88	70.88	38.79	89.63	44.70
2022	80.75	40.55	96.83	50.01	64.4	32.26	71.53	38.00	88.36	42.47
2023	85.34	42.93	99.95	51.34	70.5	35.71	72.08	38.94	100.69	46.97
AAPC (%)	2.295	-0.003	1.254	-1.350	4.078	2.429	0.217	-0.415	3.549	0.346
95% CI (%)	(1.430~3.168)	(-0.809~0.811)	(0.370~2.145)	(-2.003~-0.662)	(2.981~5.186)	(1.150~3.723)	(-0.930~1.377)	(-1.302~0.480)	(2.743~4.362)	(-0.504~1.202)
*t*	5.95	-0.01	3.17	-4.36	8.41	4.26	0.37	-1.04	9.94	0.91
*P*	<0.01	0.99	0.01	<0.01	<0.01	<0.01	0.71	0.33	<0.01	0.39

ASIR, age-standardised incidence rate; AAPC, average annual percentage change.

As shown in **Table 2**, the crude mortality rate of lung cancer in Shandong has been stable from 2012 to 2023. After adjusting the age structure, there was an obvious declined trend exhibited both in males and females, urban and rural areas. The overall ASMR reduced by 2.911% per year (AAPC=-2.911%, *P*<0.01). In addition, the ASMR in males decreased from 44.75 per 100,000 to 35.09 per 100,000 (AAPC=-2.513%, *P*<0.01), while the ASMR in females decreased from 20.41 per 100,000 to 14.54 per 100,000 (AAPC=-3.632%, *P*<0.01). The ASMR in urban and rural areas reduced from 28.17 per 100,000 and 35.69 per 100,000 to 21.41 per 100,000 and 27.14 per 100,000, respectively (Urban AAPC=-3.267%, *P*<0.01; Rural AAPC=-2.603%, *P*<0.01).

**Table 2:** Mortality of lung cancer from 2012 to 2023 in Shandong (per 100,000).

Year	Overall		Male		Female		Urban		Rural	
	Crude rate	ASMR	Crude rate	ASMR	Crude rate	ASMR	Crude rate	ASMR	Crude rate	ASMR
2012	50.46	32.00	66.10	44.75	34.54	20.41	45.69	28.17	54.99	35.69
2013	55.44	34.64	72.62	48.40	37.93	22.02	53.83	32.41	56.75	36.52
2014	57.93	34.47	75.71	48.08	39.76	21.94	56.17	32.04	59.37	36.55
2015	55.85	32.00	73.86	45.24	37.44	19.93	56.72	29.81	55.40	33.34
2016	56.82	30.83	75.48	43.80	37.77	18.93	58.61	29.84	55.51	31.63
2017	57.94	31.15	77.55	44.32	37.95	18.99	55.31	28.33	59.71	33.17
2018	57.04	29.77	76.45	42.54	37.30	17.98	55.18	27.30	58.58	32.05
2019	61.02	30.79	80.67	43.10	41.08	19.37	54.98	26.82	64.32	33.28
2020	60.68	29.67	80.87	42.15	40.03	18.11	57.35	27.11	63.11	31.63
2021	51.62	24.6	69.91	36.1	33.08	14.31	43.42	21.45	60.34	27.75
2022	56.62	25.96	76.73	38.09	36.19	15.1	49.56	23.64	62.45	27.79
2023	53.27	24.22	71.39	35.09	34.87	14.54	44.12	21.41	63.87	27.14
AAPC (%)	0.207	-2.911	0.499	-2.513	-0.362	-3.632	-0.605	-3.267	1.301	-2.603
95% CI (%)	(-0.910~1.337)	(-3.932~-1.879)	(-0.586~1.596)	(-3.404~-1.614)	(-1.572~0.863)	(-4.856~-2.392)	(-3.564~2.444)	(-4.572~-1.944)	(0.653~1.952)	(-3.404~-1.796)
*t*	0.41	-6.23	1.02	-6.18	-0.66	-6.45	-0.39	-5.45	4.49	-7.12
*P*	0.69	<0.01	0.33	<0.01	0.52	<0.01	0.69	<0.01	<0.01	<0.01

ASMR: age-standardised mortality rate, AAPC: average annual percentage change.

### Age-period-cohort analysis of the lung cancer incidence and mortality

We compared the residual deviance of different sub-models after including age, period and cohort factors, and then selected the best model to predict the future incidence and mortality of lung cancer. **Tables 3**, **4** showed the change in deviance in the sequential building of the models. Results showed that the deviance value of the age-period-cohort model (APC) for lung cancer incidence was 1217.01, indicating a good fit of the model compared with the age-cohort model (1817.48 for AC) and age-period model (1880.92 for AP). The APC model was also fitted to male, female, urban and rural populations. The deviance value of the APC model (827.12) for mortality was significantly better than the AC model (1084.97) and the AP model (1578.57). Therefore, our subsequent estimations were based on the APC model.

**Table 3:** Comparison of age-period-cohort sub-models for lung cancer incidence.

Terms in model	Overall		Male		Female		Urban		Rural	
	Residual deviance	*P* value	Residual deviance	*P* value	Residual deviance	*P* value	Residual deviance	*P* value	Residual deviance	*P* value
Age	2474.99	NA	1345.45	NA	2762.91	NA	1903.13	NA	1337.31	NA
Age-drift	2426.36	<0.01	896.39	<0.01	2493.83	<0.01	1778.76	<0.01	1336.05	<0.01
Age-cohort	1817.48	<0.01	737.68	<0.01	1168.78	<0.01	1134.54	<0.01	1195.14	<0.01
Age-period-cohort	1217.01	<0.01	493.93	<0.01	738.80	<0.01	816.27	<0.01	894.09	<0.01
Age-period	1880.92	<0.01	661.76	<0.01	2121.19	<0.01	1525.88	<0.01	1041.07	<0.01

N/A, Not applicable.

**Table 4:** Comparison of age-period-cohort sub-models for lung cancer mortality.

Terms in model	Overall		Male		Female		Urban		Rural	
	Residual deviance	*P* value	Residual deviance	*P* value	Residual deviance	*P* value	Residual deviance	*P* value	Residual deviance	*P* value
Age	3654.31	NA	2168.83	NA	1704.27	NA	2190.91	NA	1785.74	NA
Age-drift	1863.62	<0.01	1170.01	<0.01	899.89	<0.01	1183.34	<0.01	1026.16	<0.01
Age-cohort	1084.97	<0.01	568.34	<0.01	666.32	<0.01	760.42	<0.01	679.24	<0.01
Age-period-cohort	827.12	<0.01	466.47	<0.01	510.28	<0.01	674.01	<0.01	510.05	<0.01
Age-period	1578.57	<0.01	1052.77	<0.01	731.05	<0.01	1084.93	<0.01	848.09	<0.01

N/A, Not applicable.

**Figure 1:**
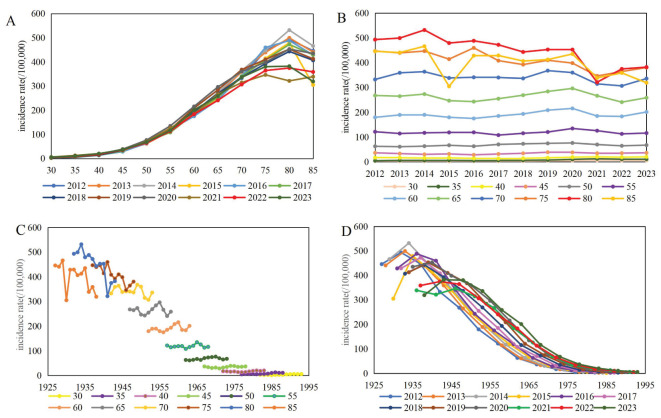
Incidence of lung cancer per 100,000 by age, period and cohort effect [**(A)** age trend by period; **(B)** period trend by age; **(C)** cohort trend by age; **(D)** cohort trend by period].

**Figure 2:**
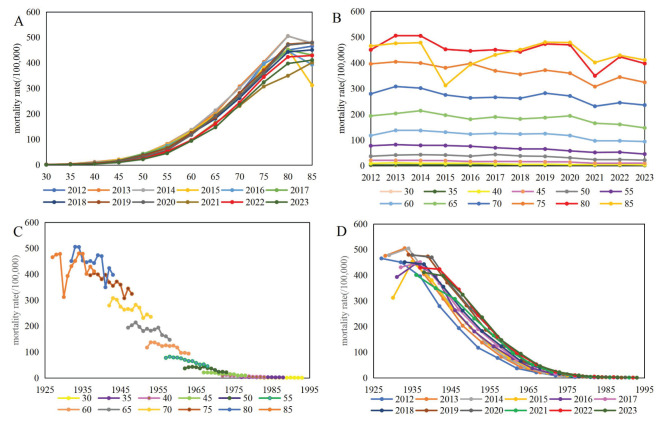
Mortality of lung cancer per 100,000 by age, period and cohort effect [**(A)** age trend by period; **(B)** period trend by age; **(C)** cohort trend by age; **(D)** cohort trend by period].

We plotted the crude incidence and mortality rates of lung cancer by age, period and cohort effects. **Figures 1**, **2** illustrated the observed crude rates in 1-year period and 5-year age group, excluding the age group under 30 years old because of the rare cases. The incidence rates increased with age in every period, rising substantially after aged 55 years, peaking at aged 80 years, and decreasing slightly aged 85 years (**Figure 1A**). During the period of 2012-2023, the incidence rates remained relatively stable among age groups under 75 years, while it decreased with the period among age groups above 75 years (**Figure 1B**). Cohort trends suggested that the cohort effect increased across age groups but diminished sharply within each period (**Figures 1C, D**). From 2012 to 2023, the mortality rates increased with age in every period, and displayed a fluctuating downward trend for each age group (**Figures 2A, B**). The cohort effect increased across age groups and decreased sharply within each period (**Figures 2C, D**).

### Predicted lung cancer incidence and mortality in Shandong, 2024-2030

**Figure 3:**
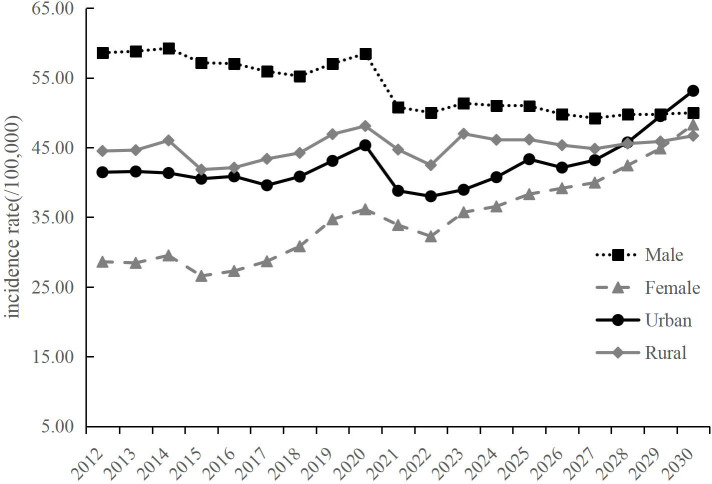
Predicted lung cancer incidence of Shandong from 2024 to 2030, by sexes and regions.

**Figure 4:**
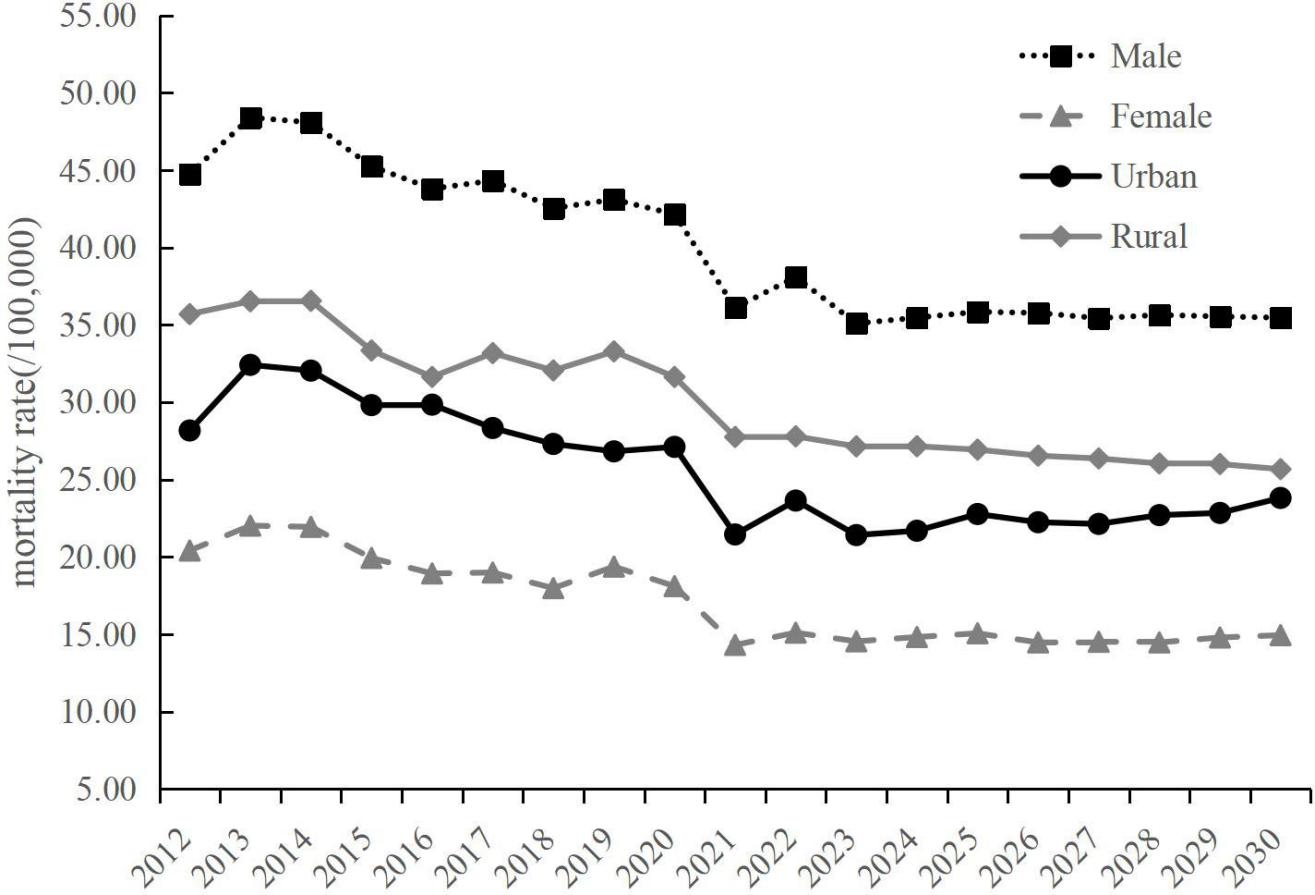
Predicted lung cancer mortality of Shandong from 2024 to 2030, by sexes and regions.

We predicted the ASIR and ASMR from 2024 to 2030 using the BAPC model, stratified by sexes and regions. The ASIR of lung cancer will increase from 43.38 per 100,000 in 2024 to 49.21 per 100,000 in 2030 (AAPC=1.873%, *P*=0.02) (**Table 5**). An upward trend is expected for females and urban areas, with the AAPC of 4.496% (*P*<0.01) and 4.176% (*P*<0.01), respectively. No significant change is observed for males and rural areas (**Table 5**; **Figure 3**). The ASMR of lung cancer in the overall population, encompassing both males and females, are expected to maintain stability up to the year 2030 (**Table 6**). An upward trend is expected for urban areas with the AAPC of 1.100% (*P*=0.03). However, for rural areas, the ASMR showed a slightly downward trend with the AAPC of -0.915% (*P*<0.01) (**Table 6**; **Figure 4**).

**Table 5:** Incidence of lung cancer from 2024 to 2030 in Shandong predicted by BAMP (per 100,000).

Year	Overall	Male	Female	Urban	Rural
2024	43.38 (38.95~48.34)	51.01 (46.19~56.21)	36.55 (31.04~42.45)	40.74 (35.20~47.20)	46.10 (40.89~51.79)
2025	44.70 (38.46~51.82)	50.95 (44.63~58.17)	38.31 (31.05~47.14)	43.32 (35.73~53.05)	46.14 (39.49~53.96)
2026	43.73 (36.48~52.28)	49.77 (42.50~58.54)	39.16 (30.88~49.87)	42.12 (33.64~54.12)	45.33 (37.53~54.80)
2027	43.91 (35.73~54.18)	49.22 (41.21~59.30)	39.96 (30.12~53.16)	43.18 (33.53~58.10)	44.82 (36.16~55.61)
2028	45.66 (36.32~57.82)	49.74 (40.70~60.95)	42.43 (30.46~58.35)	45.73 (34.68~63.16)	45.56 (35.89~57.57)
2029	46.95 (36.37~61.05)	49.80 (39.72~62.33)	44.86 (31.31~65.55)	49.50 (35.68~72.39)	45.86 (34.98~59.63)
2030	49.21 (37.17~65.64)	49.97 (39.19~63.70)	48.29 (32.80~74.31)	53.13 (37.07~80.77)	46.68 (35.10~62.27)
AAPC (%)	1.873	-0.385	4.496	4.176	0.109
95% CI (%)	(0.291~3.481)	(-1.013~0.247)	(3.588~5.413)	(1.560~6.859)	(-0.503~0.724)
*t*	2.32	-1.20	9.87	3.15	0.35
*P*	0.02	0.23	<0.01	<0.01	0.73

AAPC, average annual percentage change.

**Table 6:** Mortality of lung cancer from 2024 to 2030 in Shandong predicted by BAMP (per 100,000).

Year	Overall	Male	Female	Urban	Rural
2024	24.38 (20.89~28.72)	35.46 (30.24~41.03)	14.82(12.26~17.92)	21.69 (17.22~27.43)	27.15 (23.87~30.95)
2025	24.82 (20.02~30.78)	35.84 (28.74~44.05)	15.06(11.61~19.67)	22.77 (16.87~31.39)	26.93 (22.70~32.12)
2026	24.21 (18.57~31.61)	35.77 (27.77~46.30)	14.47(10.58~19.79)	22.24 (15.34~33.64)	26.54 (21.72~33.02)
2027	23.94 (17.81~32.33)	35.43 (26.12~47.60)	14.51(10.1~21.03)	22.13 (14.36~35.30)	26.36 (20.97~33.64)
2028	23.94 (17.27~33.80)	35.64 (25.63~49.48)	14.47(9.82~21.98)	22.70 (14.02~37.83)	26.04 (20.19~34.19)
2029	24.07 (16.70~35.10)	35.54 (24.63~50.82)	14.79(9.44~23.09)	22.84 (13.36~40.30)	26.00 (19.55~34.92)
2030	24.34 (16.20~36.54)	35.46 (23.61~52.85)	14.94(9.31~24.23)	23.81 (13.42~44.58)	25.67 (18.94~35.47)
AAPC (%)	-0.276	-0.073	-0.043	1.100	-0.915
95% CI (%)	(-0.870~0.321)	(-0.301~0.155)	(-0.907~0.829)	(0.137~2.073)	(-1.062~-0.769)
*t*	-1.19	-0.82	-0.13	2.94	-15.98
*P*	0.29	0.45	0.90	0.03	<0.01

AAPC, average annual percentage change.

## Discussion

In 2023, China has launched the plan of “Healthy China Cancer Prevention and Control Action Implementation (2023-2030)”. The plan states that by 2030, the rising incidence and mortality of cancer in China will be curbed, and the disease burden of patients will be effectively controlled. Only by understanding the future development trend of different cancers, we can evaluate the effect of current prevention and control measures and adjust the future prevention and control policies. Shandong is the second most populous province in China, with 102 million people. Lung cancer has the highest incidence and mortality rates of all cancers, in both China and Shandong province. Therefore, our study aimed to analyze and predict the development trend of lung cancer in Shandong, providing data reference for realizing the goal of “Healthy China 2030” to curb the incidence and mortality of lung cancer and optimizing the prevention and control strategy of lung cancer in the future.

In this study, we examined the lung cancer incidence and mortality trend from 2012 to 2023 in Shandong Province. We observed the crude incidence rate of lung cancer displayed an obvious upward trend but the crude mortality rate of lung cancer did not change significantly. However, after adjusting the age structure, the incidence rate remained stable while the mortality rate reduced by 2.911% per year during the 12-year period. These results were in consistent with the outcomes of a nationwide study (5). Other provinces have followed a similar trend. In developed Shanghai, the ASIR of lung cancer increased significantly with an APC of 5.12% from 2010 to 2016, while the ASMR decreased with an APC of 0.87% (34). From 2006 to 2015, the incidence and mortality of lung cancer in Jiangsu Province showed an obvious upward trend, with an average annual increase rate of 4.06% and 3.95% respectively. However, after adjusting the age structure, they tend to be stable (11). In Henan Province, the ASIR and ASMR of lung cancer showed a stable trend during 2010-2019 (10). These results could be attributed to several advancements. With the implementation of anti-smoking policies and environmental pollution control, the ASIR has been effectively controlled and is gradually becoming stable. Improvements in lung cancer treatment, the establishment of early diagnosis and treatment programs, and advancements in lung cancer screening technology have likely contributed to better patient survival rates and a reduction in mortality. In 2009, lung cancer was included in the “Rural Cancer Early Diagnosis and Early Treatment Project”, which initiated the screening of high-risk population of lung cancer in China (35). The urban cancer early diagnosis and treatment program, launched in 2012, also encompasses lung cancer screening initiatives (36). These programs have utilized low-dose spiral CT scans for lung cancer screen. Studies indicates that low-dose spiral CT has been effective in enhancing the early diagnosis rate of lung cancer, subsequently leading to a decrease in mortality rates (37).

Most developed countries, including the UK, the United States, Australia and Canada, male lung cancer incidence showed a stable or even continuous decline. These countries have a smoking epidemic earliest, so the incidence of lung cancer had been high for a long time. They adopted the tobacco control measures relatively early, and as smoking rates fell, so did lung cancer mortality (38). The United States is the most typical country, where smoking rates among men have fallen from 42% in 1990 to 13.7% in 2018 over the past 25 years, resulting in a 45% reduction in male lung cancer mortality (39). However, due to the increasing and aging population, the incidence of lung cancer will continue to increase in the near future, which is a major public health challenge. China is one of the countries with the fastest aging population growth in the world, therefore, lung cancer prevention in our country faces more challenges (40). The successful progress in the prevention and treatment of lung cancer in developed countries such as the United States can be used as a reference for the formulation of lung cancer prevention and treatment strategies in our country (41).

A marked gender disparity was found in the disease burden of lung cancer in Shandong, with men experiencing much higher incidence and mortality rates than female. Additionally, the ASIR in males decreased by 1.35% during the past 12 years, while the ASIR in females increased by 2.43%. The ASMR for both males and females have exhibited a decline, with women experiencing a more pronounced reduction of 3.63% compared to men at 2.51%. These findings corresponded with worldwide observations and our country’s data. In global, the world-standard incidence rate of lung cancer was higher in males compared to females over the period from 1990 to 2019. However, the gender gap is progressively diminishing, with a 12.5% reduction for males and a 22.3% increase for females (42). In China, the world-standard incidence rate for lung cancer among men has remained stable or slightly decreased after 2000, while it has increased by approximately 1.0% per year for women (5). It is probable that the gender difference in smoking prevalence accounts for the higher incidence and mortality in males. The persistence of smoking over time is identified as the most influential factor in determining the risk of lung cancer for smokers (43). According to the China Smoking Hazards Report 2020, approximately 296 million are men among the 308 million smokers, while the smoking prevalence for women has consistently been lower (44). In Shandong, the smoking prevalence among men (58.07%) was obviously higher than that among women (1.53%) (45). Therefore, the decline in lung cancer rates among men is largely attributed to effective tobacco control measures. Yet, the increased incidence rate in women is associated with certain specific risk factors, including exposure to secondhand smoke and cooking oil fumes (46, 47). Moreover, advancements in early detection and treatment have intensified the decrease in lung cancer mortality, contributing to prolonged survival rates.

Our results showed that the lung cancer incidence and mortality were higher in rural areas than in urban areas after adjusting the age structure. The higher incidence rate in rural areas may be associated to the lifestyle and environmental factors, particularly the urban-rural divide in the utilization of solid fuels and domestic water resources (48). In addition, the age-standardised mortality rates were on the downward trend both in urban and rural areas in our study, which was largely due to the implementation of effective tobacco control strategies and the inclusion of lung cancer screening in early diagnosis and treatment program.

Through an age-period-cohort model, we were able to determine the effects of age, period, and cohort on cancer incidence and mortality, representing an essential initial step in understanding the disease’s causal mechanisms. The extent of exposure to the vast majority of risk factors increases with age, so that almost all cancer incidence and mortality are positively associated with age (49). The period effect comprises a range of factors that concurrently affect all individuals during a particular time in history such as pollution or healthcare interventions (50). The cohort effect derives from a population-specific experience or exposure in a birth cohort, such as child malnutrition or changing habits during wartime (51, 52). The results showed that age was the key factor of lung cancer incidence and mortality, and the risk of lung cancer and death increased with age, this may be due to body’s cumulative exposure to carcinogens and increased mutations over time (53, 54). We also found the age effect was predominantly observed in the elderly population, which could be associated with the increased aging population in China. The period effect in our study showed that the risk of death from lung cancer decreased over time in all age groups. It may be attributed to the enforcement of various cancer prevention and control policies. The cohort effect could be related to an elevated educational level and a greater awareness of the disease prevention and control within the more recent birth cohorts (55). Additionally, after the establishment of the People’s Republic of China, the national economy developed steadily, the living environment of the residents improved significantly, and the medical resources continued to expand, leading to a reduction in the risks of lung cancer occurrence and mortality.

The BAPC model offered reliable and stable estimations for disease prediction (33). We predicted the incidence and mortality of lung cancer in Shandong Province from 2024 to 2030 using the BAPC model. Our results show that the ASIR of lung cancer will increase to 49.21 per 100,000 until 2030, with the AAPC of 1.873%. This upward trend is expected for females and urban areas, with the AAPC of 4.496% and 4.176%, while it is not observed for males and rural areas. The ASMR of lung cancer is expected to remain stable up to 2030, and this trend will maintain both in males and females. For urban areas, the ASMR will exhibit an increasing trend with the AAPC of 1.100%, and in contrast, it will show a slightly decreasing trend with the AAPC of -0.915% for rural areas. With the escalation of the aging population, a steady growth in lung cancer incidence is anticipated throughout the population. It is necessary to take further preventive measures such as strengthening tobacco control, enhancing health education and expanding screening efforts. Additionally, the stabilisation of lung cancer mortality after 2024 year may be mainly influenced by the incidence and survival rate of lung cancer. An increase in incidence may lead to an increase in mortality, while an increase in survival may lead to a decrease in mortality. The results of this study predicts that it shows an upward trend in the ASIR of lung cancer after 2024 year, which may be associated with increased exposure to risk factors, whereas our previous study showed that (56), the relative survival rate of lung cancer increased from 17.6% in 2012-2014 to 24.4% in 2018-2020, mainly due to the improvement of treatment level and the implementation of early diagnosis and treatment program. Therefore, the ASMR of lung cancer tends to be stable with the increase of the ASIR and survival rate.

For the prediction of outcomes in this study we need to be aware that underreporting or diagnostic errors may occur during cancer registration, and therefore part of the results may be underestimated (57). Furthermore, as this study is based on historical data over a short period of time, estimates of future rates should not be overinterpreted. The prediction of cancer burden is the basis of many epidemiological studies, which can provide scientific guidance for cancer prevention and control. Therefore, it is urgent to carry out the research of cancer burden prediction based on the more extensive coverage, more representative data, longer and more complete historical data.

In summary, the age-standardised incidence rate of lung cancer will increase until 2030 in Shandong, while the age-standardised mortality rate of lung cancer is expected to remain stable. The findings will offer valuable insights for a comprehensive understanding of the prevailing lung cancer landscape in Shandong, supplying vital information for healthcare professionals in disease surveillance and control initiatives.

## Data Availability

The raw data supporting the conclusions of this article will be made available by the authors, without undue reservation.

## References

[1] World Health Organization. Global cancer burden growing, amidst mounting need for services (2024). Available online at: https://www.who.int/news/item/01-02-2024-global-cancer-burden-growing–amidst-mounting-need-for-services (accessed February 1, 2024).

[2] Wang SM Zheng RS Han BF Li L Chen R Sun KX. Age distribution of cancer incidence and mortality in China in 2022. China Cancer. (2024) 33:165–74. doi: 10.11735/j.issn.1004-0242.2024.03.A001

[3] GBD 2015 Tobacco Collaborators. Smoking prevalence and attributable disease burden in 195 countries and territories, 1990-2015: A systematic analysis from the Global Burden of Disease Study 2015. Lancet. (2017) 389:1885–906. doi: 10.1016/S0140-6736(17)30819-X PMC5439023 28390697

[4] Siegel RL Miller KD Fuchs HE Jemal A. Cancer statistics, 2022. CA Cancer J Clin. (2022) 72:7–33. doi: 10.3322/caac.21708 35020204

[5] Li X Gao S. Trend analysis of the incidence, morbidity and mortality of lung cancer in China from 1990 to 2019. Chin J Prev Contr Chron Dis. (2021) 29:821–6. doi: 10.16386/j.cjpccd.issn.1004-6194.2021.11.005

[6] Ma JX Guo XL Fu ZT. Shandong cancer registry annual report 2021. Shandong Sci Technol Press. (2022), 34–42.

[7] National Cancer Center. China Cancer Registry Annual Report 2021. Beijing: People’s Medical Publishing House (2023). p. 84–6.

[8] National Center for Chronic and Non-communicable Disease Control and Prevention Chinese Center for Disease Control and Prevention. The atlases for the main causes of death of Chinese population. Beijing: China Cartographic Publishing House (2016) p. 89–91.

[9] National Office for Cancer Prevention and Control National Central for Cancer Registry Disease Prevention and control Bureau Ministry of Health China. Chinese cancer mortality report, the third national retrospective survey of death by cause. Beijing: People’ s Medical Publishing House (2010) p. 24–36.

[10] Guo XL Chen Q Xu HF Liu Y Wang XY Kang RH. Epidemiological characteristics of lung cancer in Henan province in 2019 and its trend from 2010 to 2019. China Cancer. (2024) 33:358–65. doi: 10.11735/j.issn.1004-0242.2024.05.A004

[11] Wang LC Zhou JY Han RQ Lu Y Luo PF Miao WG. Incidence and mortality of lung cancer in Jiangsu province in 2015 and the trend of changes during 2006-2015. China Cancer. (2020) 29:579–85. doi: 10.11735/j.issn.1004-0242.2020.08.A004

[12] Hu F Ma Y Kang H. Death trends and age-period-cohort model analyses of lung cancer, Sichuan, 2007-2021. Modern Prev Med. (2024) 51:1370–6. doi: 10.20043/j.cnki.MPM.202401393

[13] Lu CX Ma JX Ma JH Zhou H Xue JJ Ding GH. Epidemiological characteristics of lung cancer incidence in the tumor registration area of Gansu Province from 2010 to 2019. Chin J Lung Cancer. (2024) 27:88–95. doi: 10.3779/j.issn.1009-3419.2024.102.05 PMC10918250 38453439

[14] Liu J Li ZJ Yan W Xu Y Chen XN. Time trend analysis of lung cancer prevalence and disease burden in cancer registration areas in Jiangxi province. Chin J Health Statistics. (2023) 40:730–7. doi: 10.11783/j.issn.1002-3674.2023.05.022

[15] Liu ZQ Jiang YF Fang QW Yuan HB Cai N Suo C. Future of cancer incidence in Shanghai, China: Predicting the burden upon the ageing population. Cancer Epidemiol. (2019) 60:8–15. doi: 10.1016/j.canep.2019.03.004 30878799

[16] Lin X Bloom MS Du ZC Hao YT. Trends in disability-adjusted life years of lung cancer among women from 2004 to 2030 in Guangzhou, China: A population-based study. Cancer Epidemiol. (2019) 63:101586. doi: 10.1016/j.canep.2019.101586 31522131

[17] Moller H Fairley L Coupland V Okello C Green M Forman D. The future burden of cancer in England: incidence and numbers of new patients in 2020. Br J Cancer. (2007) 96:1484–8. doi: 10.1038/sj.bjc.6603746 PMC2360166 17473821

[18] Oisen AH Parkin DM Sasieni P. Cancer mortality in the United Kingdom: projections to the year 2025. Br J Cancer. (2008) 99:1549–54. doi: 10.1038/sj.bjc.6604710 PMC2579704 18854832

[19] Pesola F Ferlay J Sasieni P. Cancer incidence in English children, adolescents and young people: past trends and projections to 2030. Br J Cancer. (2017) 117:1865–73. doi: 10.1038/bjc.2017.341 PMC5729467 29096400

[20] Smittenaar CR Petersen KA Stewart K Moitt N. Cancer incidence and mortality projections in the UK until 2035. Br J Cancer. (2016) 115:1147–55. doi: 10.1038/bjc.2016.304 PMC5117795 27727232

[21] Liu SZ Zhang F Quan PL Lu JB Liu ZC Sun XB. Time trends of esophageal cancer mortality in Linzhou city during the period 1988-2010 and a Bayesian approach projection for 2020. Asian Pac J Cancer Prev. (2012) 13:4501–4. doi: 10.7314/apjcp.2012.13.9.4501 23167368

[22] Chen WQ Zheng RS Zeng HM. Bayesian age-period-cohort prediction of lung cancer incidence in China. Thorac Cancer. (2011) 2:149–55. doi: 10.1111/j.1759-7714.2011.00062.x 27755842

[23] World Health Organization. International statistical classification of diseases and related health problems 10th version (2019). Available online at: https://icd.who.int/browse10/2019/en.

[24] National Cancer Center. Guideline for Chinese Cancer Registration Vol. 2016. Beijing: People’s Medical Publishing House (2016). p. 147–62.

[25] Bray F Parkin DM. Evaluation of data quality in the cancer registry: principles and methods part I: comparability, validity and timeliness. Eur J Cancer. (2009) 45:747755. doi: 10.1016/j.ejca.2008.11.032 19117750

[26] Krishnamoorthy Y Rajaa S Giriyappa DK Bharathi A Velmurugan B Ganesh K. Worldwide trends in breast cancer incidence from 1993 to 2012: Age-period-cohort analysis and joinpoint regression. J Res Med Sci. (2020) 25:98. doi: 10.4103/jrms.JRMS_708_19 33273943 PMC7698377

[27] Ilic M Ilic I. Cancer mortality in Serbia, 1991-2015: an age-period cohort and joinpoint regression analysis. Cancer Commun (Lond). (2018) 38:10. doi: 10.1186/s40880-018-0282-3 29764495 PMC5993142

[28] Schmid VJ Held L. Bayesian age-period-cohort modeling and prediction-BAMP. J Stat Software. (2007) 21:1–15. doi: 10.18637/jss.v021.i08

[29] Zhu GQ Liu QO. Mathematical statistics of medicine. Beijing: Higher Educ Press. (2006).

[30] Berzuini C Clayton D. Bayesian analysis of survival on multiple time scales. Stat Med. (1994) 13:823–38. doi: 10.1002/sim.4780130804 8047738

[31] Yao Y Stephan KE. Markov chain Monte Carlo methods for hierarchical clustering of dynamic causal models. Hum Brain Mapp. (2021) 42:2973–89. doi: 10.1002/hbm.25431 PMC8193526 33826194

[32] Harrington SM Wishingrad V Thomson RC. Properties of markov chain monte carlo performance across many empirical alignments. Mol Biol Evol. (2021) 38:1627–40. doi: 10.1093/molbev/msaa295 PMC8042746 33185685

[33] Bray F Møller B. Predicting the future burden of cancer. Nat Rev Cancer. (2006) 6:63–74. doi: 10.1038/nrc1781 16372017

[34] Dou JM Wu CX Pang Y Bao PP Wang CF Gong YM. The incidence and mortality of lung cancer in 2016 and their trends from 2002 to 2016 in Shanghai. Tumor. (2023) 43:266–76. doi: 10.3781/j.issn.1000-7431.2023.2206-0436

[35] Zhou QH Fan YG Wu N Huang WC Wang Y Li L. Demonstration program of population based lung cancer screening in China: Rationale and study design. Thorac Cancer. (2014) 5:197–203. doi: 10.1111/1759-7714.12078 26767001 PMC4704303

[36] Dai M Shi JF Li N. Project design and target of early diagnosis and treatment of urban cancer in China. Chin J Prev Med. (2013) 47:179–82. doi: 10.3760/cma.j.issn.0253-9624.2013.02.018

[37] Wei M Qiao Y. Progress of lung cancer screening with low dose helical computed tomography. Chin J Lung Cancer. (2020) 23:875–82. doi: 10.3779/j.issn.1009-3419.2020.101.40 PMC7583869 32791651

[38] Xiao JL Zheng Y. The global prevalence and prevention progress of lung cancer. China Oncol. (2020) 30:721–5. doi: 10.19401/j.cnki.1007-3639.2020.10.001

[39] Creamer MR Wang TW Babb S Cullen KA Day H Willis G. Tobacco product use and cessation indicators among adults-United States, 2018. MMWR Morb Mortal Wkly Rep. (2019) 68:1013–9. doi: 10.15585/mmwr.mm6845a2 PMC6855510 31725711

[40] Xia C Dong X Li H Cao MM Sun DQ He SY. Cancer statistics in China and United States, 2022: profiles, trends, and determinants. Chin Med J (Engl). (2022) 135:584–90. doi: 10.1097/CM9.0000000000002108 PMC8920425 35143424

[41] Schilsky RL Nass S Le Beau MM Benz EJ Jr. Progress in cancer research, prevention, and care. N Engl J Med. (2020) 383:897–900. doi: 10.1056/NEJMp2007839 32877579

[42] GBD 2019 Respiratory Tract Cancers Collaborators. Global, regional, and national burden of respiratory tract cancers and associated risk factors from 1990 to 2019: a systematic analysis for the Global Burden of Disease Study 2019. Lancet Respir Med. (2021) 9:1030–49. doi: 10.1016/S2213-2600(21)00164-8 PMC8410610 34411511

[43] Doll R Peto R Boreham J Sutherland I. Mortality in relation to smoking: 50 years’ observations on male British doctors. BMJ. (2004) 328:1519. doi: 10.1136/bmj.38142.554479.AE 15213107 PMC437139

[44] National Health Commission of the People’s Republic of China. The National Health Commission released “Report on health hazards of smoking in China, 2020”. Available online at: https://www.gov.cn/xinwen/2021-05/30/content_5613994.htm (accessed May 30, 2021).

[45] Li JL Qiao YJ Deng LP Dong HL Wu BY. Status of smoking, secondhand smoke exposure and cognition of tobacco harm in patients with chronic diseases in Shandong province. Med J Chin People’s Health. (2018) 30:89–91. doi: 10.3969/j.issn.1672-0369.2018.07.040

[46] Jiang DM Zhang LJ Liu WB Ding YB Yin JH Ren RB. Trends in cancer mortality in China from 2004 to 2018: A nationwide longitudinal study. Cancer Commun (Lond). (2021) 41:1024–36. doi: 10.1002/cac2.12195 PMC8504142 34251754

[47] Qiu AY Leng SG McCormack M Peden DB Sood A. Lung effects of household air pollution. J Allergy Clin Immunol Pract. (2022) 10:2807–19. doi: 10.1016/j.jaip.2022.08.031 36064186

[48] Yu K Qiu GK Chan KH Lam KB Kurmi OP Bennett DA. Association of solid fuel use with risk of cardiovascular and all-cause mortality in rural China. JAMA. (2018) 319:1351–61. doi: 10.1001/jama.2018.2151 PMC5933384 29614179

[49] Wong IOL Schooling CM Cowling BJ Leung GM. Breast cancer incidence and mortality in a transitioning Chinese population: current and future trends. Br J Cancer. (2015) 112:167–70. doi: 10.1038/bjc.2014.532 PMC4453599 25290086

[50] Nasreen S Wilk P Mullowney T Karp I. Age, period, and cohort effects on asthma prevalence in Canadian adults, 1994–2011. Ann Epidemiol. (2020) 41:49–55. doi: 10.1016/j.annepidem.2019.11.005 31874791

[51] O’Brien RM. Mixed models, linear dependency, and identification in age-period-cohort models. Stat Med. (2017) 36:2590–600. doi: 10.1002/sim.7305 28378504

[52] Rutherford MJ Lambert PC Thompson JR. Age-period-cohort modeling. Stata J. (2010) 10:606–27. doi: 10.1177/1536867X1101000405

[53] Peto R Parish SE Gray RG. There is no such thing as ageing, and cancer is not related to it. IARC Sci Publ. (1985) 58):43–53. 3830884

[54] Campisi J d’Adda di Fagagna F. Cellular senescence: when bad things happen to good cells. Nat Rev Mol Cell Biol. (2007) 8:729–40. doi: 10.1038/nrm2233 17667954

[55] Rees PH. Education’s role in China’s demographic future. Proc Natl Acad Sci USA. (2021) 118:e2115618118. doi: 10.1073/pnas.2115618118 34625495 PMC8590466

[56] Jiang F Fu ZT Lu ZL Chu J Guo XL Xu AQ. Cancer survival during 2012-2018 in cancer registries of Shandong Province. Chin J Prev Med. (2022) 56:806–14. doi: 10.3760/cma.j.cn112150-20210910-00882 35785863

[57] Wei WQ Zeng HM Zheng RS Zhang SW An L Chen R. Cancer registration in China and its role in cancer prevention and control. Lancet Oncol. (2020) 21:e342–9. doi: 10.1016/S1470-2045(20)30073-5 32615118

